# Comparative effectiveness of gamified binocular treatment versus conventional patching for amblyopia: a randomized clinical trial

**DOI:** 10.3389/fmed.2025.1560203

**Published:** 2025-06-02

**Authors:** Ying Chen, Yuzhen Chen, Xiao Han, Zijian Yang

**Affiliations:** Department of Ophthalmology, Sunshine Rehabilitation Center, Tongji University School of Medicine, Shanghai, China

**Keywords:** amblyopia, binocular therapy, dichoptic treatment, stereopsis, home-based treatment

## Abstract

**Introduction:**

Amblyopia, a prevalent visual disorder in children, leads to deficits in visual acuity, stereoacuity, and daily functioning. Conventional treatments such as eye patching, often encounter adherence challenges and relapse, especially in younger patients. This study investigates the effectiveness of a home-based, gamified binocular therapy (GBT), compared with traditional patching.

**Methods:**

A randomized controlled trial was conducted with 42 participants aged 4–8 years diagnosed with amblyopia from Sunshine Rehabilitation Center, Shanghai. Participants were randomly assigned to the GBT (*n* = 20) or the patching group (*n* = 22). Visual acuity (VA) and stereoacuity improvements were assessed at baseline, 1, 3, and 6 months.

**Results:**

Both groups demonstrated significant VA and stereoacuity improvements from baseline at 1 month. GBT participants exhibited faster initial VA improvement, with significant gains at 1 month compared to patching (mean difference = 0.072 logarithm of the minimum angle of resolution[logMAR], *p* = 0.0029). By 6 months, both groups showed similar improvements in VA (GBT: 0.19 ± 0.10 logMAR; patching: 0.23 ± 0.16; *p* = 0.156) and stereoacuity (GBT: 0.95 ± 0.76 log arcseconds; patching: 0.82 ± 0.59; *p* = 0.712). No significant adverse events were reported in either group.

**Conclusion:**

The study indicates that GBT is as effective as patching in improving amblyopic visual outcomes among Chinese children 4 to 8 years of age. These findings suggest that gamified binocular therapy could play an important role in personalized amblyopia treatment, with further studies needed to confirm long-term effects across diverse populations.

## Introduction

Amblyopia is a global health concern with a prevalence of 1 to 5% worldwide (1). The prevalence varies by region, age, ethnicity, race, and other factors, e.g., the prevalence in Europe is higher than in Africa (2). Amblyopia is associated with significant deficits in visual function (3), including reduced contrast sensitivity (4, 5), impaired fixation stability (6), diminished depth perception (7), and disrupted binocular vision (8), potentially influencing daily life activities (9–11). Encouraging the use of the weaker eye through methods such as occlusion of the stronger eye by patching (12), atropine eye drops (13), or applying penalizing optical filters (14) after the refractive correction and adaptation (15) is widely adopted as a conventional management strategy in the clinic. The effect of this widely used approach is solid across populations, although multiple limitations were also shared, e.g., low compliance with patching protocols (with adherence reported between 44 and 57%) (16, 17), incomplete resolution of visual deficits (18), and a relapse rate of approximately 25% even after successful treatment (19). To address these limitations and enhance treatment adherence, binocular amblyopia therapy using dichoptic presentation has been developed over the past decade (20). Hess et al. (21) developed several binocular therapy and demonstrated that dichoptic training, using a Tetris game on an iPod with low contrast to the fellow eye, improved visual acuity and stereoacuity in amblyopic adults, supporting the efficacy of binocular therapy for amblyopia (22). Recently, CureSight, a novel investigational digital dichoptic device for home-based binocular treatment of amblyopia that involves passive viewing of video content, is as effective as traditional patching (23–25). However, the long-term benefits (over 3 months) of dichoptic devices with active visual tasks, such as games, remain underexplored compared to patching in clinical settings, with conflicting results. Kelly et al. (26) investigated the short-term (4 weeks) treatment effect of an iPad game and compared it with patching; they found that the iPad game was effective in treating childhood amblyopia and was more efficacious than patching at the 2-week visit. Conversely, Yao et al. (27) reported that patching was more effective than binocular games in Chinese children. Suwal et al. (19) investigated the effect of active vision therapy in the clinic and found similar effectiveness compared to the patching group. However, the active vision group in their study also performed patching at home, leaving the independent effectiveness of active tasks unclear. To validate the true impact of binocular training with active tasks, we developed an alternative gamified treatment system, the Vision Planet System, and explored its effectiveness in individuals of Chinese ancestry. The evaluation was carried out through a randomized controlled trial comparing the new system to conventional amblyopia treatment over 6 months.

## Materials and methods

### Study design

This prospective, evaluator-masked, randomized controlled trial was conducted at Sunshine Rehabilitation Center, Shanghai. All participants underwent treatment under the supervision of ophthalmologists. The study adhered to the principles of the Declaration of Helsinki and received ethical approval from the institutional review board or ethics committee at the participating institution (2022-041-AMD-01). Written informed consent was obtained from all participants’ parents or guardians, who were informed of their right to withdraw from the study at any point. A total of 42 participants were enrolled, with 37 having completed the study. Efficacy outcomes were assessed at baseline and 1, 3, and 6 months following the treatment. This study was prospectively registered in the Chinese Clinical Trial Registry (ChiCTR), an official WHO-recognized primary registry, under the registration number ChiCTR2300067763. The trial protocol, objectives, eligibility criteria, and outcome measures were submitted prior to participant enrollment and are publicly accessible at: https://www.chictr.org.cn/showprojEN.html?proj=189576. The registration complies with the International Committee of Medical Journal Editors (ICMJE) guidelines for transparency in clinical trial conduct and reporting.

### Participants

Participants diagnosed with amblyopia were recruited from Sunshine Rehabilitation Center, Shanghai. The enrollment period started on 1 March 2023, and the last participant completed the 6-month follow-up on 7 June 2024. Key inclusion criteria were as follows: (1) age between 4 and 8 years; (2) diagnosed with amblyopia caused by strabismus, anisometropia, or both; (3) a visual acuity difference of two lines or more between the two eyes; (4) visual acuity in the amblyopic eye between 20/32 and 20/200; and (5) for children aged 4–5 years, best-corrected visual acuity (BCVA) in the dominant eye of 20/40 or better, and for those aged 5 years and above, BCVA of 20/32 or better. Exclusion criteria were as follows: (1) any anatomically documented ocular abnormalities; (2) a history of ocular surgery or concurrent conditions affecting BCVA; and (3) communication or cognitive impairments that would prevent cooperation during the study. The detailed inclusion and exclusion criteria are listed in Table 1.

**Table 1:** Eligibility criteria.

**Category**	**Specific criterion**
Inclusion criteria	Participants were eligible for inclusion in the study based on the following criteria: 1 Age 4-8 years; 2 Diagnosed with amblyopia caused by strabismus, anisometropia, or both; 3 **Criteria for strabismus amblyopia**: Diagnosed with amblyopia, determined by investigator that strabismus potential underlying cause. The presence of heterotropia observed during examination at either distance or near fixation. (with or without optical correction, up to 10 PD by SPCT). **Criteria for Anisometropic Amblyopia (met any one of criteria)** Difference in spherical equivalent ≥ 1.5 D between the two eyes, A difference in astigmatism ≥ 1.00D between corresponding axes of the two eyes. **Criteria for combined-mechanism amblyopia**: Diagnosed with strabismus ≥1.5 D difference between eyes in SE or ≥ 1.00 D difference in astigmatism between the corresponding meridians in the 2 eyes 4 A visual acuity difference of 2 lines or more between the two eyes; 5 Visual acuity in the amblyopic eye ranging from 20/32 to 20/200; 6 For children aged 4-5 years, best-corrected visual acuity (BCVA) in the dominant eye of 20/40 or better, and for children aged 5 years or older, BCVA of 20/32 or better; 7 No other binocular treatment received within 15 days prior to enrollment; 8 No other organic ocular conditions identified; 9 Informed consent obtained, with approval from the ethics committee at the hospital.
Exclusion criteria	Participants were excluded from the study if they met any of the following criteria: Allergic reaction to patch adhesive; Anatomically documented ocular abnormalities; History of ocular surgery or concurrent conditions causing reduced BCVA; Communication or cognitive disorders preventing compliance with the study protocol; History of epilepsy; Withdrawal from treatment before completion. (6) Withdrawal from treatment before completion.
Withdrawl criteria	Participants were withdrawn from the study under the following conditions: Voluntary withdrawal of informed consent; Occurrence of an adverse event (AE), or withdrawal due to concerns regarding the risk of AEs; Voluntary discontinuation of participation; Termination of the study due to policy changes or other external factors; Other force majeure events leading to study withdrawal.
Dropout documentation	For any participant who exited the study, the exact time and reason for dropout was documented in detail.

BCVA, best-corrected visual acuity; D, diopter; logMAR, logarithm of the minimum angle of resolution; PD, prism diopter; SE, spherical equivalent; SPCT, simultaneous prism and cover test; VA, visual acuity.

### Vision planet training system

**Figure 1:**
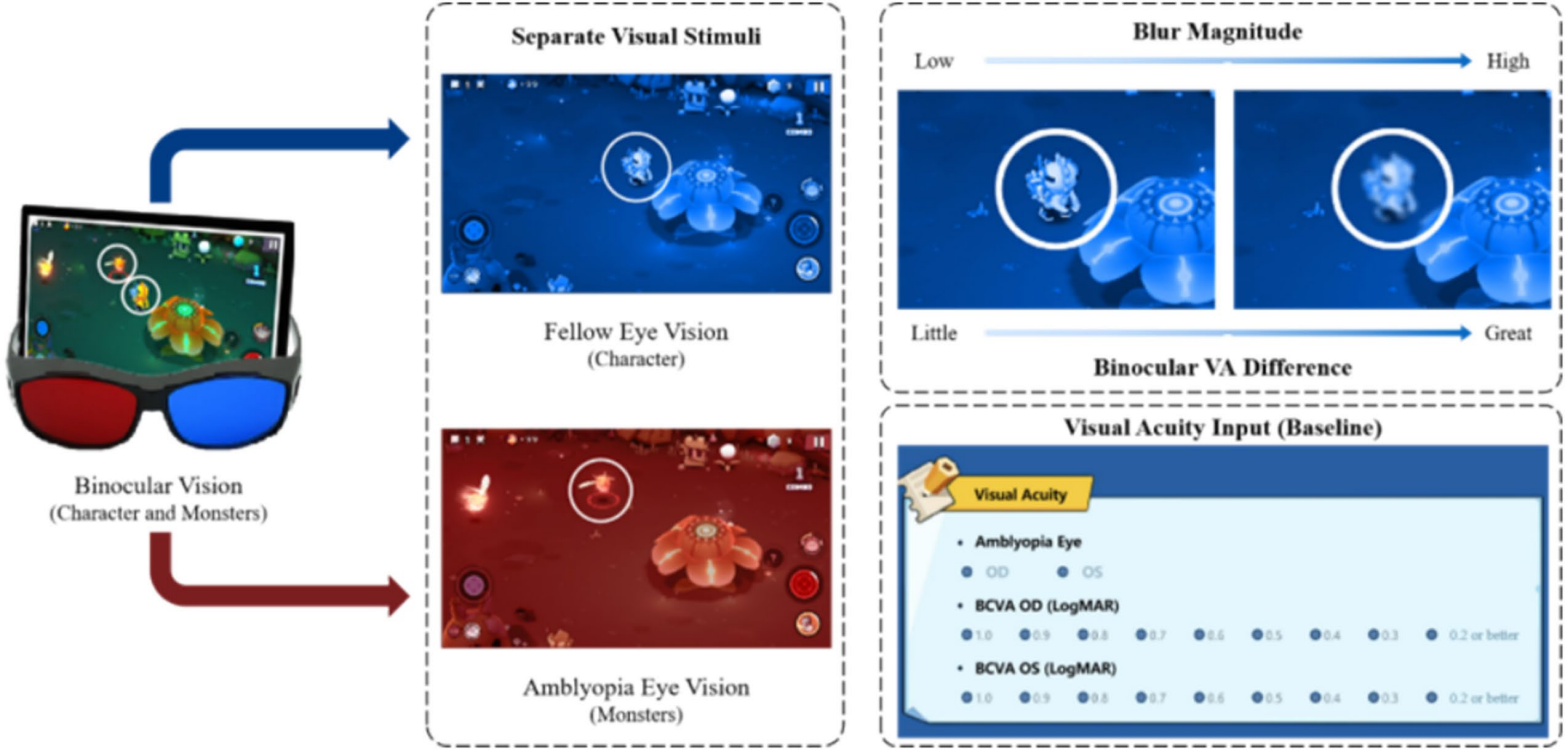
Overview of Vision Planet Training System. The figure illustrates the binocular treatment setup: (1) Training stimuli are provided through two separate channels, delivering different visual content to each eye. “Characters” and “monsters” are presented in blue and red to the amblyopic eye and fellow eye. (2) The blur intensity is automatically adjusted based on the initial acuity difference between the eyes, with greater blur applied for larger differences. (3) A training report is automatically generated at the end of each session.

The Vision Planet Training System (VPTS) is a digital therapeutic software designed to deliver visual stimuli through two independent channels, presenting similar but distinct visual content to each eye (Figure 1). The system incorporates a Roguelike shooting game element(characterized by randomly generated elements such as items, enemies, and level layouts, making them accessible to new players and offering high flexibility in contents), aiming at the improvement of visual acuity in the amblyopia eye and enhance stereoacuity, which was certified as a Class II medical device by the National Medical Products Administration (NMPA) of China (Identifier: zhexiezhuzhun20212210379).

The system includes the following components: (1) an 11-inch MatePad (model DBR-W00, screen resolution 2,560 × 1,600 and fresh rate 120 Hz) for presenting visual stimuli, (2) stereoscopic glasses were worn throughout the treatment to separate visual stimuli presented to each eye, and (3) a gamified therapeutic software named “Vision Planet.” The software automatically adjusts the blur intensity based on the baseline binocular differences in visual acuity, applying greater blur for larger differences. Participants’ baseline information (e.g., name, age, uncorrected visual acuity, best-corrected visual acuity, and refractive error) is entered into the system before treatment. The system initiates with a monocular enhancement mode for the amblyopic eye. Binocular training mode is activated once the visual acuity difference between the amblyopic and fellow eye is reduced to less than two lines, and stereoacuity training is introduced when best-corrected visual acuity exceeds 20/40 on the Snellen scale. During training, participants use a virtual joystick and attack button to control character movements and attack monsters, aiming to achieve simultaneous fixation and binocular fusion. Besides, VPTS incorporates a real-time artificial intelligence (AI) visual engine that continuously monitors and prompts participants to maintain an approximate 40 cm viewing distance from the screen and ensures proper use of the complementary stereoscopic glasses throughout the session to standardize the intervention (Figure 1).

At the core of VPTS is a blur modulation algorithm, which dynamically adjusts the Gaussian blur applied to the dominant (fellow) eye based on the baseline interocular visual acuity difference. A larger acuity gap results in increased blur intensity to reduce suppression and encourage binocular integration. In the early training stages, the system initiates a **monocular enhancement mode**, in which only the amblyopic eye is stimulated with clear, high-contrast visuals, while the fellow eye is suppressed. This mode transitions into binocular training once the interocular acuity difference is reduced to less than two lines. When visual acuity improves to better than 20/40, the system introduces stereoacuity training tasks, requiring real-time depth discrimination.

During gameplay, visual targets (“monsters”) are rendered primarily to the amblyopic eye, while controllable avatars (“characters”) are directed to the fellow eye. Users interact via a virtual joystick and shooting panel, facilitating simultaneous fixation, fusion, and vergence adaptation. Training modules include fine motor training (via character control and target tracking), fusion tasks (requiring the combination of disparate stimuli into coherent scenes), and stereoacuity challenges (using depth-based object placement and movement).

A real-time AI-based visual monitoring engine continuously tracks participant compliance. The engine evaluates viewing distance (~40 cm), eye alignment, and stereoscopic glasses usage. If improper use is detected, such as uncalibrated eye position or missing glasses, the system automatically suspends the session until proper conditions are restored.

Additional visual enhancement mechanisms include embedded red-light stimulation (wavelength 630–750 nm) during specific in-game scenes, and grating stimuli based on the Cambridge Visual Stimulator (CAM) integrated into visual effects, such as resurrection animations and bonus levels. These modules aim to activate cortical neurons and enhance macular fixation. A parental dashboard and backend system log session frequency, duration, and performance metrics to support treatment adherence monitoring.

### Procedure

The participants were randomly allocated to either the control group (receiving patching) or the binocular group (receiving VPTS) by a randomization sequence generated by R software version 4.2.0. All participants underwent cycloplegic refraction and completed at least 8 weeks of refractive adaptation with stable visual acuity in the amblyopic eye (changes in VA less than 1 line). For the binocular group, the training sessions were 30 min each, conducted twice daily. For the patching group, each session lasted 90 min and was conducted once daily. Both groups followed this regimen 7 days a week for 6 months.

Participants in the binocular group underwent home-based treatment using VPTS, which included various training modules such as fine motor training, fusion training, and stereoacuity training. Fine motor training focuses on enhancing eye-hand coordination by requiring participants to control a game character using a virtual joystick for navigation and an attack button to eliminate visual targets (“monsters”). This task necessitates continuous gaze tracking and precise spatial coordination, thereby strengthening visual-motor integration—a critical skill often impaired in amblyopic children. Fusion training is implemented through the dichoptic display of asynchronous visual elements: the amblyopic eye views distinct targets (e.g., monsters), while the fellow eye perceives the controllable avatar or environmental obstacles. Participants must coordinate inputs from both eyes to successfully interact with in-game elements, fostering simultaneous fixation and sensory fusion under binocular conditions. Stereoacuity training becomes available once participants reach specific visual acuity thresholds (i.e., best-corrected acuity in the amblyopic eye is better than 20/40). In these levels, depth-based visual cues—such as floating platforms, layered enemy placement, or depth-variant rewards—require discrimination of relative distances using stereoscopic vision. Polarized or anaglyph glasses are required to complete these tasks, and system-integrated checks ensure that participants are wearing them appropriately. Each training session begins with automatic system checks to confirm the correct viewing distance (approximately 40 cm) and proper glasses usage. Failure to meet these standards results in a temporary session suspension. Game progression includes tutorial, main, elite, and bonus levels that embed these training modules in varied contexts, reinforcing compliance and reducing fatigue. Collectively, these modules deliver a multimodal, immersive therapeutic experience tailored to improve both monocular and binocular function in amblyopic patients.

At the beginning of each session, participants were required to maintain an optimal distance of 40 cm from the screen. The system automatically verified whether participants were correctly wearing red-blue glasses during stereoacuity training; if the glasses were not worn properly, the system would automatically terminate the session.

Participants assigned to the control group wore an eye patch over their dominant eye for 90 min per day, 7 days a week, over 6 months. If daily patching for 90 min was not feasible, the treatment was divided into shorter sessions totaling 90 min per day. Participants were instructed to log their daily patching time using a designated link. For participants in the control group, the prescribed patching regimen consisted of 90 min of daily occlusion therapy, applied to the dominant (fellow) eye, 7 days a week for 6 consecutive months. In cases where a continuous 90-min session was not feasible—due to scheduling, behavioral compliance, or caregiver constraints—the total daily duration could be divided into multiple shorter sessions, provided that each segment lasted a minimum of 30 min. Over the 6-month intervention period (approximately 180 days), participants in the binocular training group completed two 30-min sessions per day, resulting in a theoretical total training duration of approximately 180 h (60 min/day × 180 days). In contrast, participants in the patching group were prescribed 90 min of occlusion therapy per day, yielding a cumulative total of approximately 270 h across the same period.

Outcome assessments were conducted at baseline and at 1, 3, and 6 months (±1 week) post-treatment at the hospital. Examiners performing the visual acuity and stereoacuity testing were blinded to group assignments. Distance best-corrected visual acuity (BCVA) was measured using a Lea symbols chart. Stereoacuity was assessed at 40 cm with the Titmus stereo test (Stereo Optical Company, Inc.) using polarized lenses. Treatment adherence was monitored differently across the two groups. In the binocular training group, all training activities were conducted through the Vision Planet software, which automatically recorded session duration, frequency, and completion status via its backend system. These data were accessible to caregivers through the built-in *Parent Center* and were used to monitor daily adherence objectively. In contrast, for the patching group, treatment adherence was recorded via self-reported caregiver logs, and submitted through a designated online platform. Although caregivers were instructed to log patching time in real-time, retrospective batch entries could not be entirely ruled out. As such, the accuracy of the patching adherence data may be subject to recall bias or temporal inaccuracy, and we acknowledge this as a methodological limitation of the study. Future studies may consider incorporating electronic occlusion dose monitors (e.g., occlusion dose monitors or wearable sensors) to further improve adherence tracking in patching protocols.

### Outcome

Primary and secondary outcome measures were collected for all participants at baseline and 1-, 3-, and 6-month post-treatment. The primary efficacy outcome was defined as the BCVA improvement in the amblyopic eye from baseline in both groups, measured using a Lea symbols chart, following the standardized procedures outlined in the ATS protocol manual, conducted by a certified masked examiner. The secondary efficacy outcome was defined as the improvement in stereoacuity from baseline, converted to log arcseconds. Participants who could only recognize the stereoscopic fly, or who were unable to recognize any stereoscopic figures, were assigned a value of 3,000 arcseconds.

### Statistical analysis

Mixed-model analysis of variance (ANOVA) was used to evaluate changes in BCVA over time, with individual samples treated as a random effect to account for variability among participants. Fixed effects included time, treatment type (binocular vs. patching), and their interaction. Pairwise comparisons were conducted using the student’s t-test to assess differences in treatment effects at each time point. Non-parametric tests, such as the Wilcoxon signed-rank test, were applied for stereoacuity data. Statistical significance was set at *α* = 0.05 for all analyses, and the threshold was adjusted by the number of tests if necessary (Bonferroni correction). All the analyses were performed in R software version 4.2.0.

## Result

### Demographic and baseline characteristics

**Figure 2:**
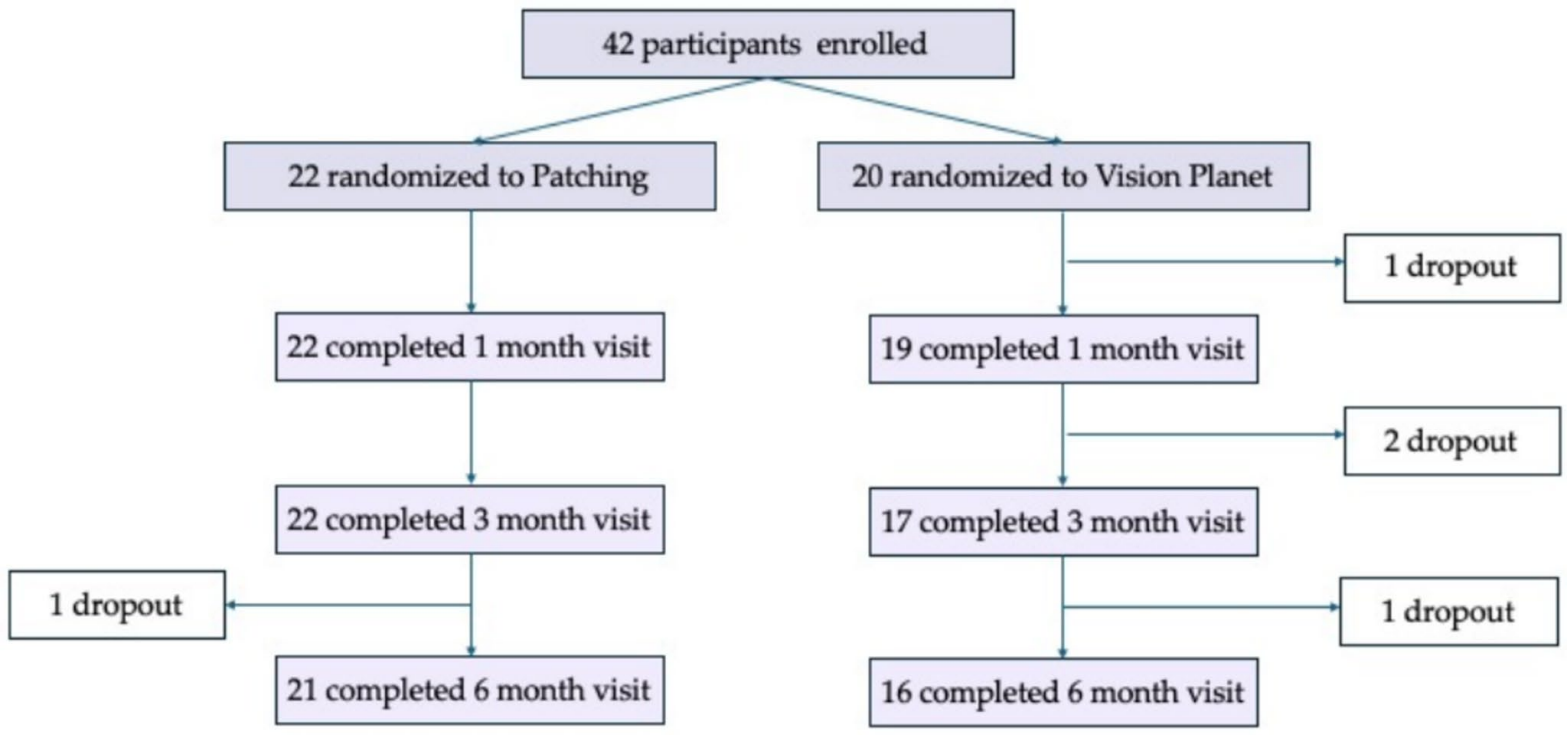
Overview of the follow-up events in samples. Longitudinal follow-up was conducted for both groups at 1-, 3-, and 6-month post-treatment. The sample dropout resulted from parents’ reluctance to follow up, as confirmed by telephone, not from participants’ discomfort.

A total of 42 participants were recruited, with 20 assigned to the binocular group and 22 to the patching control group (Figure 2). Of these, 37 participants completed the 6-month follow-up, including 16 in the binocular group and 21 in the patching group. Among the five participants who dropped out, two completed the 3-month follow-up, four attended the 1-month follow-up, and one did not participate in any follow-up visits. Dropouts occurred due to unwillingness to continue with follow-up. No adverse events, such as eye strain, headaches, worsening visual acuity, significant increase in heterotropia (≥10 prism diopters), or other severe symptoms, were observed in any participants.

Demographic and baseline characteristics were analyzed for both groups, revealing no significant differences between groups in age by independent sample t-test, gender, amblyopic eye, type of amblyopia, or baseline BCVA. Refractive amblyopia (including anisometropic amblyopia) accounted for 93%, with strabismic amblyopia and combined amblyopia comprising 4.7 and 2.3%, respectively, with no between-group differences by Fisher’s exact test. A detailed summary of the demographic characteristics is presented in Table 2.

**Table 2:** Demographics and baseline characteristics.

Characteristic	The binocular group	Patching group	*P*-value
Age(yrs)	5.60 ± 1.47	6.09 ± 1.57	0.303
Gender			
Male	6(20)	9(22)	0.461
Female	14(20)	13(22)	
Amblyopia eye			
Right eye	16(30)	20(40)	0.782
Left eye	14(30)	20(40)	
Type of amblyopia			
Refractive	17(20)	22(22)	0.813
Strabismus	2(20)	0(22)	
Both combined	1(20)	0(22)	
Baseline visual acuity (logMAR)	0.287 ± 0.140	0.372 ± 0.225	0.057

log MAR, logarithm of the minimum angle of resolution. Data are presented as no.(total), no. (%), or mean±standard deviation.

### Primary outcome

**Figure 3:**
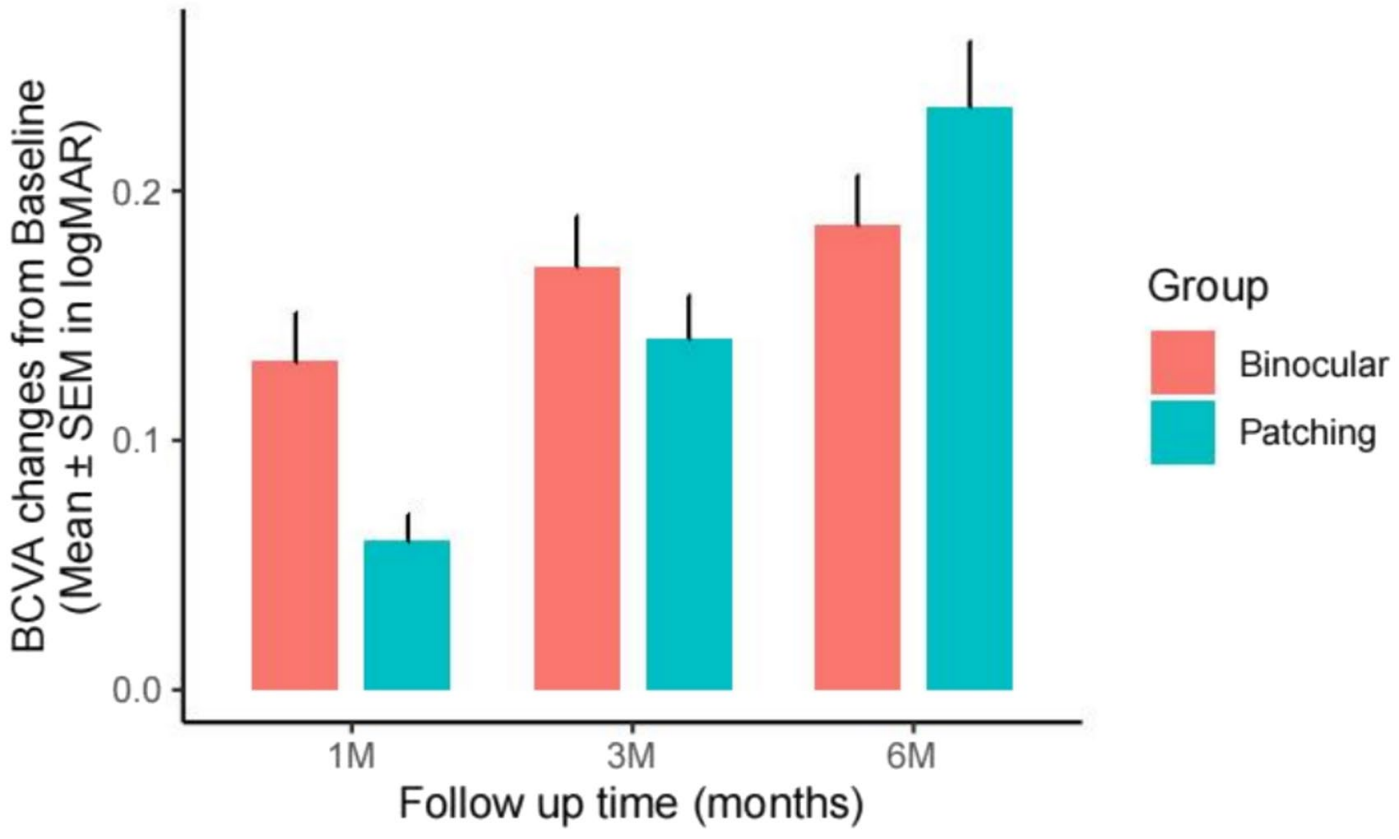
BCVA improvement from baseline at 1-, 3-, and 6-month post-treatment in the binocular and patching groups, measured in logMAR. Bar height indicates mean improvement from baseline (BCVA_time – BCVA_baseline), with error bars representing the SEM of the improvement.

Both the binocular and patching groups showed significant improvements in best corrected visual acuity (logMAR) from baseline after accounting for multiple testing. At baseline, the binocular group had a mean logMAR of 0.29 ± 0.14 and the patching group had 0.37 ± 0.23 (*t* = −1.94, *p* = 5.70 × 10^−2^). The mixed-model ANOVA, with samples as a random effect, revealed that visual acuity was significantly associated with both time (*F* = 88.04, *p* < 2.20 × 10^−16^) and treatment type (*F* = 28.86, *p* = 1.81 × 10^−7^). Additionally, there was a significant interaction effect between time and treatment type (*F* = 5.33, *p* = 1.50 × 10^−3^). Within the patching group, mean logMAR improvements from the baseline were 0.059 ± 0.068 at 1 month (*t* = 5.48, *p* = 2.97 × 10^−7^), 0.14 ± 0.11 at 3 months (*t* = 8.10, *p* = 6.89 × 10^−10^), and 0.23 ± 0.16 at 6 months (*t* = 8.91, *p* = 9.61 × 10^−11^). The binocular group showed improvements of 0.13 ± 0.11 at 1 month (t = 6.59, *p* = 4.55 × 10^−7^), 0.17 ± 0.10 at 3 months (*t* = 8.34, p = 1.50 × 10^−8^), and 0.19 ± 0.10 at 6 months (*t* = 9.30, *p* = 2.95 × 10^−9^) to the baseline. The binocular group had a greater improvement during the first month compared to the patching group (mean difference = 0.072, 95% CI: 0.026–0.118, *t* = 3.16, *p* = 2.90 × 10^−3^), but differences were not significant at 3 months (*p* = 0.285) or 6 months (*p* = 0.156), indicating a faster initial response in the binocular group, possibly due to better participant compliance (see Figure 3).

### Secondary outcome

**Figure 4:**
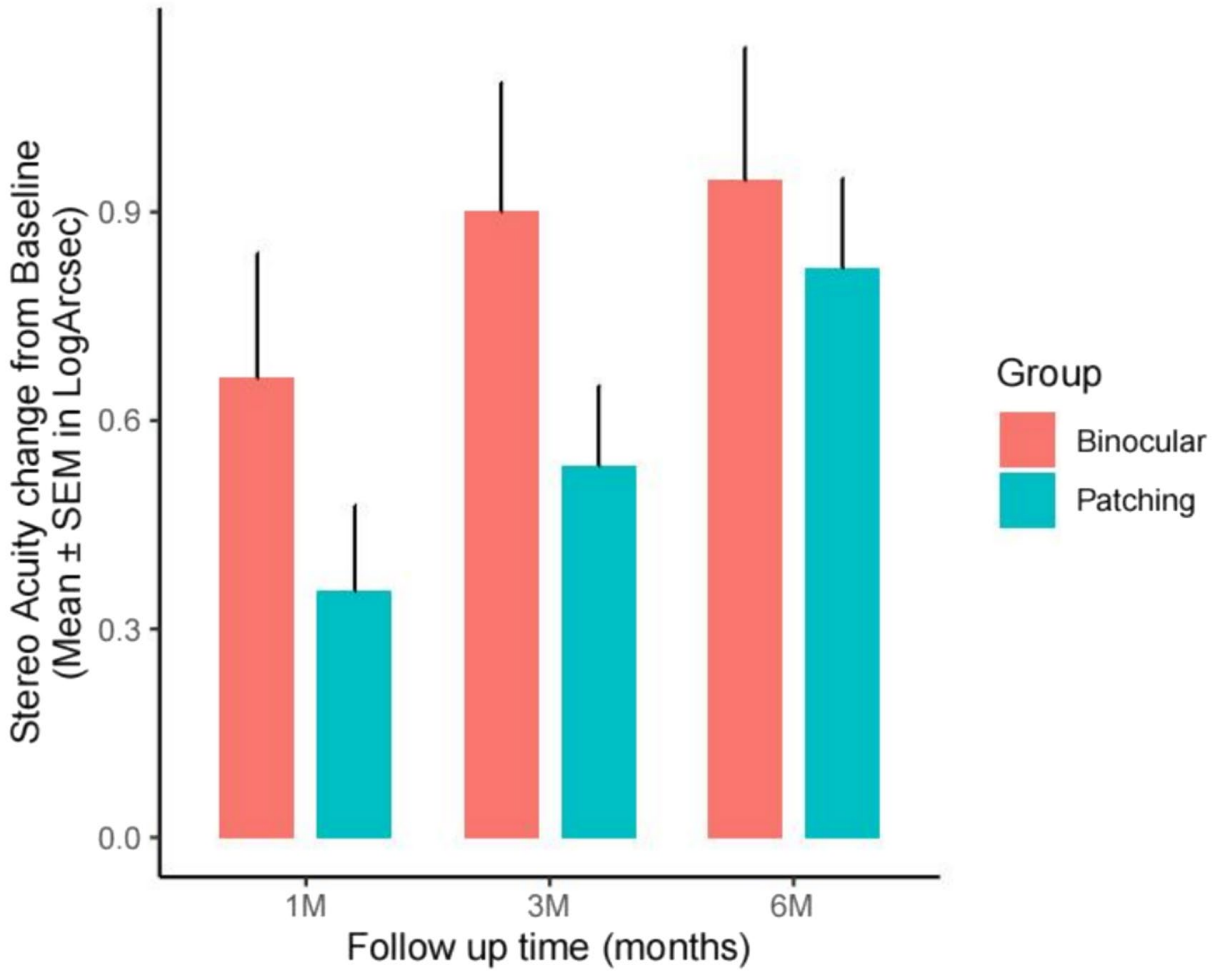
Stereoacuity (SA) improvement from baseline at 1-, 3-, and 6-month post-treatment in the binocular and patching groups, measured in log arcseconds. Bar height indicates mean improvement from baseline (SA_time – SA_baseline), with error bars representing the SEM of the improvement.

Stereo acuity (logArcsec) measured under best correction showed significant improvements from baseline in both groups, although baseline measurements were not significantly different (*W* = 161.5, *p* = 0.1332). For the binocular group, significant improvements in stereo acuity were observed at 1 month (*V* = 91, *p* = 1.65 × 10^−3^), 3 months (*V* = 120, *p* = 7.23 × 10^−4^), and 6 months (*V* = 120, *p* = 7.18 × 10^−4^) after accounting for multiple testing (adjusted alpha = 0.008). The patching group also showed significant improvements at those visit times (1 month: *V* = 45, *p* = 7.41 × 10^−3^; 3 months: *V* = 190, *p* = 1.29 × 10^−4^; 6 months: *V* = 231, *p* = 6.27 × 10^−5^). However, the differences in improvement between the two groups were not statistically significant due to high variability, with *p*-values of 0.1025, 0.05555, and 0.7123 at 1, 3, and 6 months, respectively. While the binocular group tended to show faster improvements, this trend did not reach statistical significance (see Figure 4).

To compare the effectiveness and adherence between the binocular and patching groups, satisfaction scores, collected via questionnaire, and training time records, obtained from device exports for the binocular group and parent-reported recall cards for the patching group, were analyzed. In the binocular group, 81.3% of participants reported a satisfaction score of ≥9 (on a 0–10 scale), significantly higher than the 60.9% in the patching group, indicating greater satisfaction with the binocular treatment. Compliance was assessed by calculating the deviation from the target training duration, defined as [(actual time − target time) / target time], where the target time was 180 h for the binocular group and 270 h for the patching group. The median deviation was −1.7% for the binocular group and −13.7% for the patching group, with negative values indicating that participants generally completed less than the target training duration, suggesting some degree of non-compliance. The difference in deviation was statistically significant (Wilcoxon rank sum test, *W* = 253, *p* = 0.0096), demonstrating better adherence in the binocular group compared to the patching group.

## Discussion

In a randomized controlled trial (RCT), our study found that a home-based gamified binocular treatment device achieved comparable effectiveness to patching for amblyopia in Chinese children. The binocular group showed a faster response and greater improvement compared to the patching group in the first month, then the two groups showed a similar improvement both in visual acuity and stereo acuity our study findings align with existing literature, demonstrating that binocular treatment involving active tasks yields comparable improvements in visual acuity to traditional patching therapy for amblyopia. Specifically, when compared to the passive task approach reported by Wygnanski-Jaffe et al., our binocular treatment group, using the patching group as a reference, exhibited a relatively greater improvement in visual acuity at 3 months post-treatment [0.03 logMAR (0.17–0.14) vs. -0.01 logMAR (0.22–0.23) in their study]. This suggests that active binocular tasks may enhance treatment outcomes compared to passive interventions. Furthermore, during the initial month of treatment, our binocular group demonstrated a statistically significant improvement in visual acuity (mean = 0.13 logMAR) compared to the patching group (mean = 0.06 logMAR). This indicates a faster and more robust response to gamified binocular treatment, consistent with findings from Kelly et al. (26), who also reported accelerated visual acuity gains with similar approaches. In contrast, Yao et al. (27) reported a reduced treatment effect of binocular gaming compared to patching in a Chinese population, a finding that diverges from most studies, including ours. However, by implementing AI-monitored training to optimize treatment adherence and engagement, our study achieved consistent and favorable results in a similar demographic, suggesting that advanced monitoring technologies may mitigate previously observed disparities in treatment efficacy. These results underscore the potential of active, gamified binocular treatments as a viable alternative to patching, with the added benefit of faster initial improvements in visual acuity.

Subject adherence plays a crucial role in the success of amblyopia treatment, with studies showing that only approximately 50% of caregivers maintain the recommended patching times for children (25). Higher gains in visual acuity (VA) are often linked to better adherence (28). Our study demonstrated that participants in the binocular group reported significantly higher satisfaction and better adherence compared to the patching group. The higher satisfaction scores (81.3% vs. 60.9%) and smaller deviation from the target training time (median −1.7% vs. −13.7%) suggest that binocular treatment was not only more acceptable to participants but also easier to maintain over time. This improved adherence may have contributed to the early gains in VA observed in the binocular group. These findings underscore the potential of engaging, game-based binocular therapies to enhance compliance and satisfaction, key factors for successful amblyopia treatment. In addition, in digital-based binocular treatments, training tasks can be either active or passive, although the outcome for the active and passive tasks did not differ significantly from the literature (29–37). For example, CureSight involves passive video watching for half an hour, while our study used action games that required active engagement from participants. The significant and rapid improvement observed in the binocular group during the first month may be attributed to the increased effort and engagement required by the active tasks, along with the high adherence to the training schedule observed in our study; this is also supported by the findings of Kelly et al. (26). Although the patching group showed notable VA improvement by 6 months, the difference compared to the binocular group was not statistically significant. Incorporating a variety of customizable training activities may help sustain interest and improve treatment outcomes in future study designs.

Several limitations should be considered in our study. First, the sample consisted of children aged 4–8 years with anisometropia, small-angle strabismus, or a combination of both. While these subtypes are common in clinical practice, further research is needed to evaluate the effectiveness of binocular treatment across a wider range of conditions to confirm its generalizability. Some studies have suggested that the binocular approach may yield greater improvements in older age groups, such as teenagers and adults (38, 39). Second, the sample size was relatively small. Our data have already demonstrated the effectiveness of the binocular treatment compared to the baseline. However, a larger sample from multicenter studies may still be needed to confirm the observed trends between groups in the future. Finally, the follow-up period was short, which may have limited our ability to capture long-term treatment effects. Extending the follow-up duration in future studies will be important for a more comprehensive evaluation of outcomes.

## Conclusion

Our randomized controlled trial demonstrated that a home-based gamified binocular treatment device was as effective as patching for amblyopia in Chinese children. These findings suggest that gamified binocular treatment is a viable approach for amblyopia, though further research with larger samples and longer follow-up is needed to confirm these benefits across diverse populations.

## Data Availability

The raw data supporting the conclusions of this article will be made available by the authors, without undue reservation.
