# Correction: Comparative effectiveness of gamified binocular treatment versus conventional patching for amblyopia: a randomized clinical trial

**DOI:** 10.3389/fmed.2025.1689341

**Published:** 2025-09-15

**Authors:** Ying Chen, Yuzhen Chen, Xiao Han, Zijian Yang

**Affiliations:** Department of Ophthalmology, Sunshine Rehabilitation Center, Tongji University School of Medicine, Shanghai, China

**Keywords:** amblyopia, binocular therapy, dichoptic treatment, stereopsis, home-based treatment

In the published article, Ying Chen and Yuzhen Chen were erroneously omitted as equal contributing authors. The corrected statement reads: ‘*These authors have contributed equally to this work and share first authorship.'*

The original version of this article has been updated.

